# Enhanced and tunable white light emission from Ag nanoclusters and Eu^3+^-co-doped CaBAl glasses

**DOI:** 10.1039/c8ra07114d

**Published:** 2018-10-15

**Authors:** Marcelo Sandrini, Robson Ferrari Muniz, Vitor Santaella Zanuto, Franciana Pedrochi, Yannick Guyot, Antonio Carlos Bento, Mauro Luciano Baesso, Alysson Steimacher, Antonio Medina Neto

**Affiliations:** Departamento de Física, Universidade Estadual de Maringá Maringá PR Brazil sandrini_m@hotmail.com; CCSST, PPGCM, Universidade Federal do Maranhão Imperatriz MA Brazil; Institut Lumière Matière, Université de Lyon, UMR5306 Université Lyon 1 - CNRS Villeurbanne France

## Abstract

Noble metal embedded glasses have been studied as promising candidates for a variety of technological applications, mainly due to their ability to enhance rare earth luminescence properties. In this work, Ag:Eu-co-doped calcium boroaluminate glasses were prepared and submitted to further heat treatment to form different Ag species. The optical and luminescence properties were investigated in terms of heat treatment times. Absorption spectra showed a successful Eu and Ag ion incorporation in the host, as well as Ag nanoparticle precipitation induced by heat treatment. Upon UV-light excitation, the co-doped glasses exhibited an intense wide emission band centered at about 500 nm, attributed to molecule-like silver species, which combined with the Eu^3+^ characteristic emission reaches a white light resultant emission. A new excitation band for Eu^3+^ at 335 nm and a silver luminescence lifetime decrease suggest an energy transfer process from molecule-like Ag to Eu^3+^ as being responsible for the enhanced PL properties in these glasses. An appropriate combination of a violet LED with the sample emission provides a route to achieve the ideal white light CIE color parameters. The relevant quality color results qualify these glasses as phosphors with high potential for white light emitting devices.

## Abstract 2

In this work, Ag:Eu-co-doped calcium boroaluminate glasses were prepared and submitted to further heat treatment to form different Ag species. The optical and luminescence properties were investigated in terms of heat treatment times.

## Graphical abstract

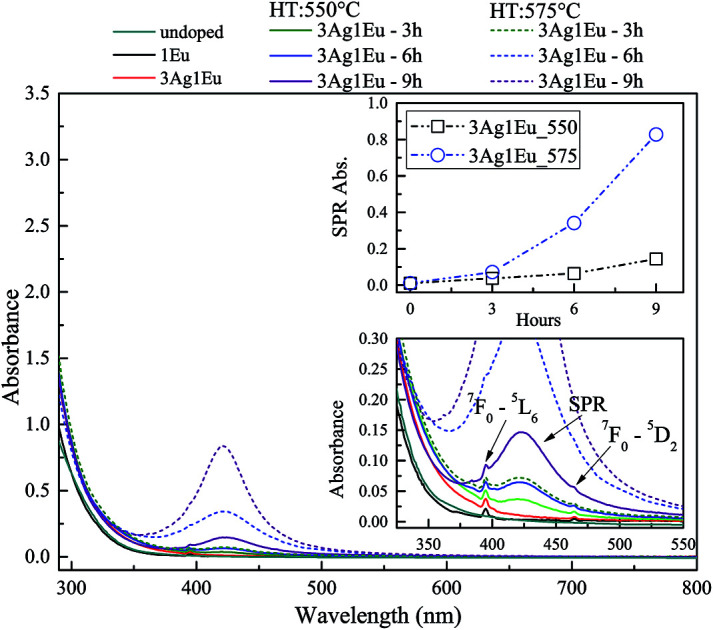

## 1 Introduction

Artificial lighting is present in all segments of society, making it crucial to develop new more efficient and more economical light emitting devices. In addition, society is increasingly demanding that technological development, in the most diverse areas, occurs in line with human well-being and health requirements. Recent studies have shown that several disturbances in the regulation of human physiology, behavior and circadian cycle can be induced by artificial light exposure.^1–3^ Therefore, smart white light has been studied, with the aim of developing highly efficient devices and a color balance control, in order to reproduce the variations of daylight spectral distribution.^4–6^

Due to the advantages of high brightness, long lifetime and low power consumption, white light (WL) LEDs have often been used to replace conventional lighting sources. These devices are based on a phosphorus element excited by an UV LED chip, and are considered the new generation of solid-state lighting devices. Furthermore, the correct combination of UV LED intensity with some phosphor emission may simulate the sunlight cycle and synchronize the human circadian rhythm, a feature of the class of materials named Smart White LEDs. The commercial WL has crystalline phosphorus (YAG: Ce^3+^, for example) usually encapsulated with an epoxy resin. As an alternative, rare earth metal (RE) doped glass phosphors have attracted large amounts of attention, due to their chemical and thermal stability, luminescent performance, low cost and easier large scale production, compared to crystalline systems.^4–10^

Many investigations have focused on the co-doping of noble metals in glass systems in order to improve the RE luminescence properties. Preliminary works in this field focus on the combination of RE elements with these transition metals. Malta *et al.*,^11^ for instance, were one of the first to report an enhancement of Eu^3+^ luminescence properties in a glass host due to the local-field surface plasmon resonance (SPR) effect from silver nanoparticles (Ag NPs). The same conclusions were obtained in subsequent works.^12–14^ On the other hand, special attention has been given to silver co-doped glass due to the fact that several Ag species can be formed during the Ag NPs precipitation processes. The clustering of Ag^0^ atoms and Ag^+^ free ions may form molecule-like silver nanoclusters (Ag NCs, also expressed as Ag^*n*+^_*m*_). Jiménez *et al.*^15^ concluded that Eu^3+^ luminescence enhancement is a result of energy transfer processes from molecule-like Ag species to Eu^3+^ ions whereas Ag NPs provide a path for the non-radiative loss of excitation energy leading to a PL quenching effect. Other reports also ascribe the RE luminescence enhancement to the energy transfer process from intermediate Ag species (isolated Ag^+^ or Ag NCs) to RE ions instead of SPR contribution.^8,16–19^ Thus, the actual interaction mechanism between Ag species and RE ions is a controversial and unclear question, which requires further significant investigation.

In addition, due to molecular fluorescence mechanisms, the presence of Ag NCs provides the host matrix with a wide luminescent band property with high quantum yields. The Ag NC emission band covers approximately all the visible range (350 to 750 nm) and it is more efficient when excited in the range of 325 to 430 nm.^8,9,20,21^ When combined with the RE luminescence properties, the Ag NC emission band may provide a broad band white light with higher quality parameters, such as high color rendering index (CRI) and low correlated color temperature (CCT).

In this context, with the aim of contributing to further understanding of the interaction between noble metals and RE ions in glass hosts, and developing new materials for artificial light emitting devices, we have studied the spectroscopic properties of calcium boroaluminate (CaBAl) glass co-doped with silver and europium.

CaBAl glass stands out due to its excellent optical and chemical properties, such as high optical transparency, low melting temperature, high thermal stability, high chemical durability and high rare earth solubility.^4,7,22^ Moreover, the incorporation of CaF_2_ in the composition leads to a phonon energy decrease in these glasses, which can improve their quantum efficiency.^4,23^

In this work, Ag:Eu-co-doped CaBAl glasses were prepared. In order to induce the different Ag species, samples were heat treated at 550 and 575 °C for different lengths of time. The photoluminescence properties were systematically studied as a function of heat treatment time. The spectroscopic results showed that tunable white light with high color parameters can be achieved. Furthermore, photoluminescence (PL) spectroscopy and decay curves reveal an efficient energy transfer process between Ag NCs (donor) and Eu^3+^ ions (acceptor) as being responsible for the luminescence enhancement, which implies that SPR does not significantly affect the emission properties.

## 2 Experimental methods

Ag/Eu singly and co-doped CaBAl glasses were synthesized *via* a conventional melt-quenching method in an air atmosphere, at 1200 °C for 120 minutes, in platinum crucibles. A detailed synthesis description is found in previous works.^4,7^ The nominal glass compositions were (25 − *y* − *x*)CaO–50B_2_O_3_–15Al_2_O_3_–10CaF_2_:*y*AgNO_3_:*x*Eu_2_O_3_, where *y* = 0, 3 wt% and *x* = 0, 1, 2.5 wt%, which are named as *y*Ag:*x*Eu when co-doped. All samples were synthesized using high purity (>99.98%) chemical materials.

In order to obtain samples with Ag NPs, heat treatments (HTs) were performed at 550 and 575 °C over time periods of 3, 6 and 9 hours. The temperatures selected were slightly below the glass transition temperature (*T*_g_ ∼ 610 °C).^22^

Optical absorption spectra were recorded with a Perkin-Elmer spectrometer model Lambda 1050 within the wavelength range 275–800 nm.

Photoluminescence spectroscopy was performed at room temperature (∼24 °C). Excitation spectra were obtained by monitoring the emission at 614 nm, related to the ^5^D_0_–^7^F_2_ transition of Eu^3+^. Luminescence spectra were measured under 335 and 405 nm excitation, using a Xe lamp as an excitation source and the emission intensity was collected by a Newport monochromator model 77 780, assembled with a photomultiplier tube, the signal from which was analyzed by a lock-in amplifier (Stanford Research System, model SR830). Emission decay curves were acquired using the same photoluminescence experimental arrangement using an OPO laser (Opotek) with a pulse width of about 10 ns and the emission signals were recorded by a digital oscilloscope Tektronix model DPO4102B. The lifetime instrumental measurement error was estimated to be 1%.

## 3 Results and discussion

### 3.1 Optical absorption

**Fig. 1:**
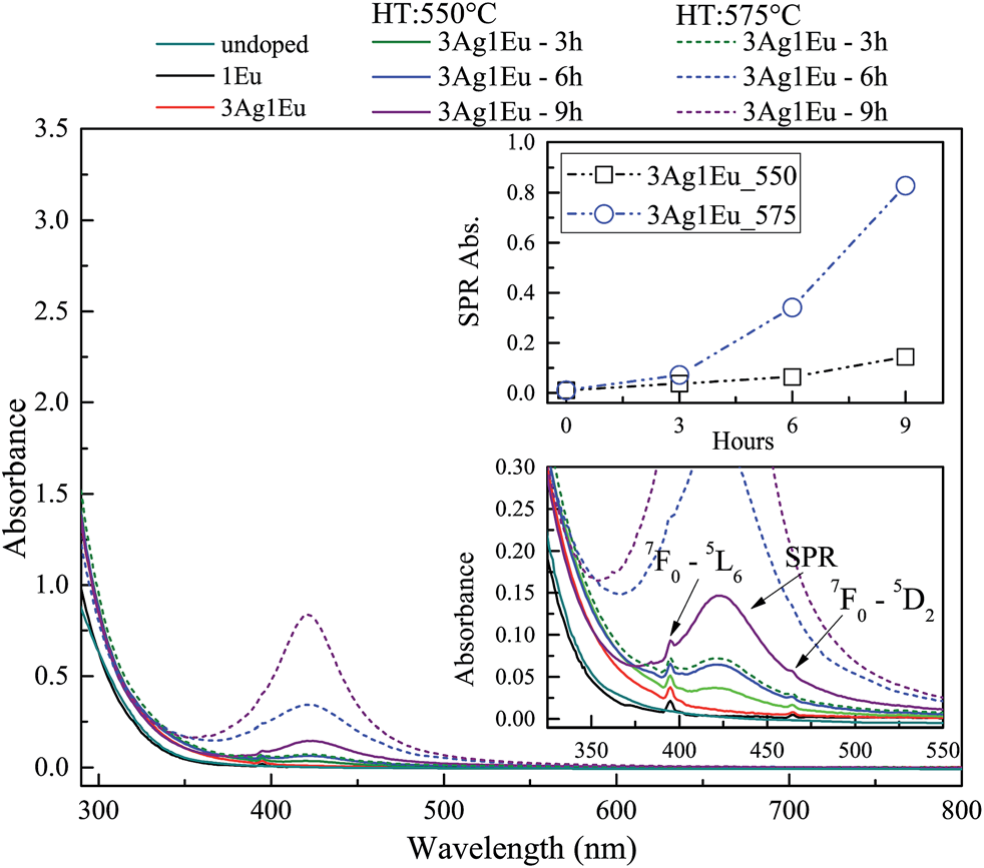
Absorbance spectra of undoped, 1Eu singly doped and 3Ag:1Eu-codoped CaBAl glasses. Codoped samples heat-treated at 550 °C (solid lines) and 575 °C (dashed). Bottom inset shows the spectra in the range of 325–550 nm. Upper inset shows the SPR absorption intensity, at 420 nm, as a function of the HT time.

**Fig. 2:**
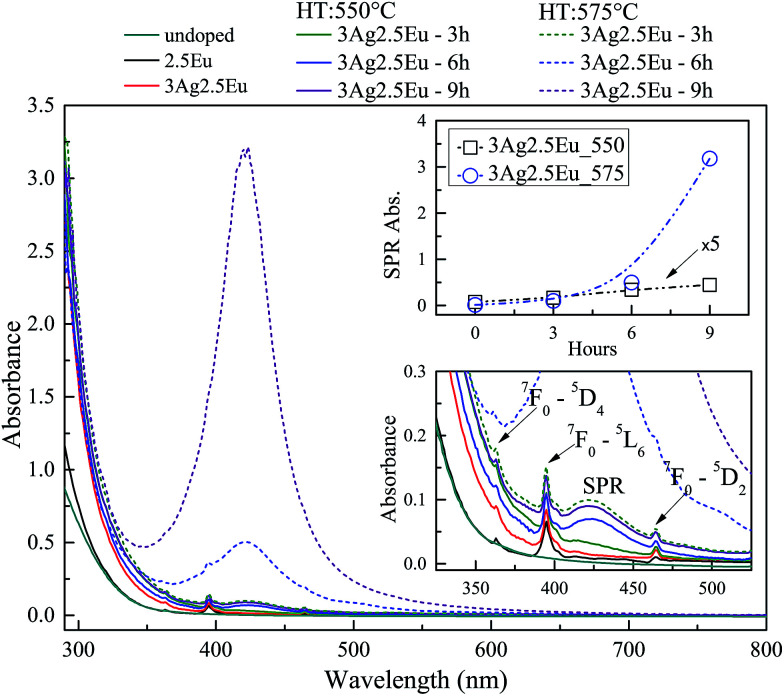
Absorbance spectra of undoped, 2.5Eu singly doped and 3Ag:2.5Eu-co-doped CaBAl glasses. Codoped samples heat-treated at 550 °C (solid lines) and 575 °C (dashed). Bottom inset shows the spectra in the range of 325–550 nm. Upper inset shows the SPR absorption intensity, at 420 nm, as a function of the HT time.

UV-Vis absorbance spectra of undoped, 1Eu singly doped and 3Ag:1Eu CaBAl glasses are presented in Fig. 1. Continuous and dashed lines show the absorption of the samples treated at 550 and 575 °C, respectively. The spectra of undoped, 2.5Eu singly doped and 3Ag:2.5Eu CaBAl glass samples are shown in Fig. 2.

In both figures, the bottom insets highlight the spectra from 325 to 550 nm, which present narrow absorption bands corresponding to Eu^3+^: 4f–4f transitions from the fundamental state ^7^F_0_ to the excited states ^5^D_4_ at 364 nm, ^5^L_6_ at 395 nm and ^5^D_2_ at 465 nm.^4,7,14^ The Ag:Eu-co-doped samples show an absorption edge red shift in the near UV range, as compared to the spectra of undoped and Eu singly doped glasses. This behavior can be ascribed to 4d^10^ → 4d^9^ 5s^1^ transitions of isolated Ag^+^ and/or S_0_ → S_1_ singlet–singlet transitions of Ag NCs (Ag^*n*+^_*m*_). The Ag^+^ transitions are forbidden in free ions, but partially allowed when inserted in glass hosts.^8,24–26^

The heat treated samples present an absorption band centered around 420 nm, which is attributed to the SPR associated with the metallic Ag NPs.^8,18,27,28^ Top insets present the SPR absorption intensity at 420 nm as a function of HT time, which increases with longer HT times and a higher temperature for both glass compositions. Furthermore, the samples doped with higher europium concentrations exhibit a higher rate for the intensity increase in SPR absorption compared to samples with a lower europium content indicating a higher density of Ag NPs, which agrees with the behavior reported by Jiao *et al.*^27^

The results indicate the incorporation of Eu and Ag ions in the host as well as the HT process promotes the precipitation of metallic Ag NPs. Additionally, the non-significant shift in the SPR band and the relatively narrow bandwidths indicate a narrow size distribution. The particles average radius was estimated to be about 5 nm by the relationship:

1 *R* = *v*_f_/Δ*ω*_1/2_

where *R* is the particle radius, *v*_f_ = 1.39 × 10^8^ cm s^−1^ is the Fermi velocity and Δ*ω*_1/2_ is the full width at half maximum (FWHM) of the SPR absorption peak. More details about this estimation method are in ref. 20 and 29.

### 3.2 Photoluminescence properties

**Fig. 3:**
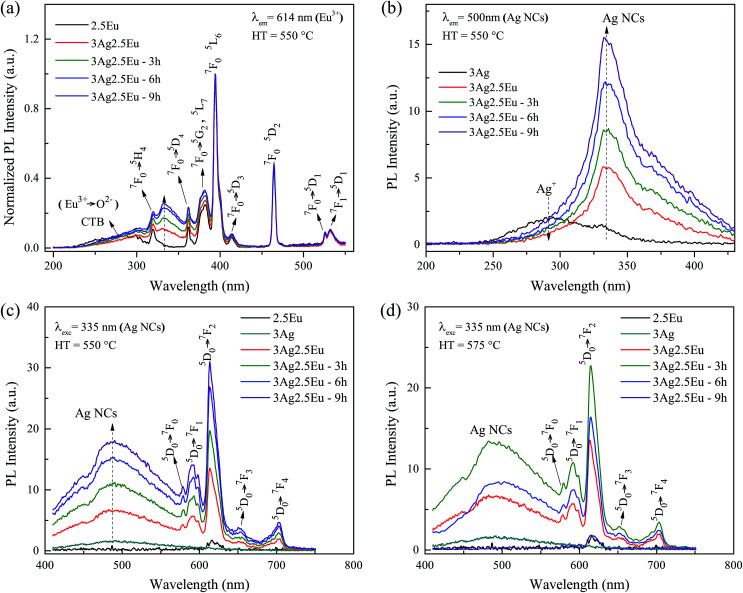
Excitation spectra of Ag:Eu CaBAl glasses heat treated at 550 °C monitoring (a) Eu^3+^ emission at 614 nm and (b) Ag NC emission at 500 nm. Emission spectra under 335 nm excitation of samples heat treated at (c) 550 °C and (d) 575 °C.

Excitation and emission spectra further confirmed the incorporation of europium ions as well as the formation of silver species in the host matrix. In Fig. 3(a), the excitation spectra of 2.5Eu singly doped and 3Ag:2.5Eu-co-doped samples are examined by monitoring the Eu^3+^ emission at 614 nm (^5^D_0_ → ^7^F_2_ transition) as a function of HT time. The spectra are composed of a broad band emission (230–315 nm), due to the well-known charge transfer band (CTB) Eu^3+^ → O^2−^, and several narrow peaks attributed to Eu^3+^: 4f–4f transitions.^9,27^ In addition, a novel excitation band centered at about 335 nm is formed with Ag introduction; this is associated with the presence of molecule-like Ag species (Ag NCs).^8,27,28^ The arising of this new excitation band suggests the possibility of efficient energy transfer (ET) from Ag NCs (donor) to Eu^3+^ ions (acceptor).

Fig. 3(b) shows the excitation spectra of 3Ag singly doped and 3Ag:2.5Eu-co-doped CaBAl glasses, monitoring the Ag NCs emission at 500 nm. The spectrum of the sample without europium is composed of a broad band ranging from 250 to 375 nm with a shoulder at 335 nm. The broad band is attributed to the Ag^+^ free ions, while the shoulder is attributed to the Ag^*n*+^_*m*_ species.^26–28^ It can be concluded that the Ag^+^ species is more favorable during the sample production process, but with Eu incorporation, the broad band decreases significantly while the shoulder becomes predominant, which suggests that the europium leads to the Ag NC formation. It is worth highlighting that the same photoluminescence behavior was observed in the 3Ag:1Eu-co-doped samples (not shown).

The emission spectra, under 335 nm excitation, are shown in Fig. 3(c) (HT at 550 °C) and Fig. 3(d) (HT at 575 °C). Under 335 nm excitation, the Eu singly doped sample presents only a low emission peak at 614 nm, characteristic of the Eu^3+^ transition. The low emission intensity is expected since europium trivalent ions have no efficient excitation at this wavelength when inserted into the CaBAl glass host.^4^ In contrast, a wide broad band centered at about 500 nm coupled with several sharp peaks in the red region, is presented by Ag:Eu-co-doped samples. The peaks are related to the Eu^3+^: ^5^D_0_ → ^7^F_*J*_ (*J* = 1, 2, 3 and 4) transitions and the broad band to silver aggregates (Ag NCs) and Eu^2+^ transitions.^4,9,14,27,28^ However, due to the peak shape, lower emission intensity compared with the Eu^3+^ emission, and the fact that the Ag singly doped sample showed the same emission centered at 500 nm, we can conclude that the broadband emission is predominantly from the Ag NCs. However, the possible Eu^2+^ and Eu^3+^ co-existence cannot be disregarded. Besides that, there is a hollow (∼464 nm) in the broad emission band suggesting a slight re-absorption by the Eu^3+^: ^7^F_0_ → ^5^D_2_ transition.

The samples HT at 550 °C, Fig. 3(c), present an increase in the emission intensities of the Ag NCs with the HT time. This result is in accordance with the excitation spectra behavior, indicating an increase of the concentration of these species. Another key point is the significant increase of the Eu^3+^ emission intensities in the co-doped samples in comparison with the spectrum of the Eu^3+^ singly doped sample, supporting the possibility of an ET process from the molecule-like Ag NCs (donor) to the europium (acceptor) in the co-doped glasses. Whereas, when annealed at 575 °C, Fig. 3(d), the maximum luminescence increase is achieved at 3 hours. Longer HT times result in a gradual luminescence decrease, reaching total suppression of Ag NC luminescence in the sample heat treated for 9 hours. This behavior may be due to the clustering of Ag aggregates and Ag^+^ free ions, which form plasmonic Ag NPs and result in luminescence re-absorption and quenching effects. This is in accordance with the high SPR absorption intensity. Jiménez *et al.*^15^ reported a similar result, an Eu^3+^ emission quenching in silver/tin doped aluminophosphate glass. This behavior was attributed to a high Ag NP precipitation, leading to a dominant Eu^3+^ → Ag NP energy transfer. In such a case, the metallic particles are acting as a plasmonic diluent.

**Fig. 4:**
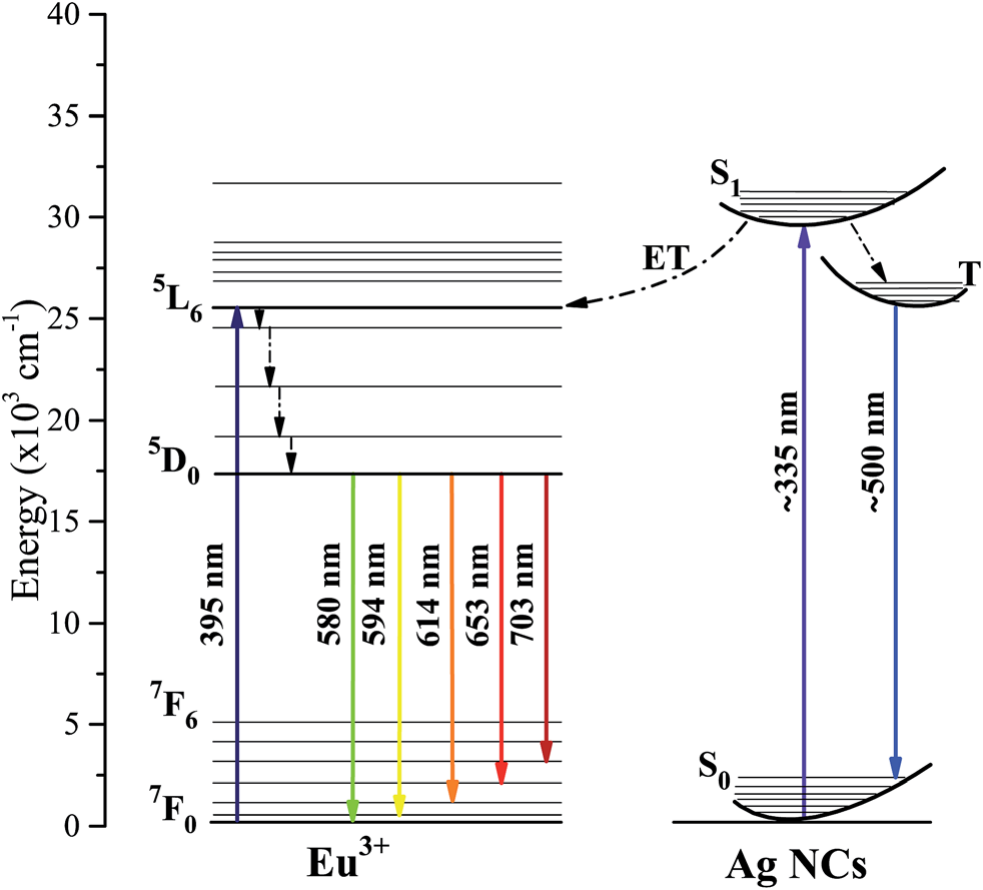
Schematic energy levels of Ag:Eu-co-doped glasses under 335 and 395 nm excitation.

A schematic energy levels diagram of Eu^3+^ and Ag NCs, with possible transition routes as well as the related ET processes, is shown in Fig. 4. Ag NCs are very sensitive to the environment coordinates, therefore, their energy states are configured as energy broad bands. On the order hand, the 5s5p orbital of Eu^3+^ ions shields the 4f energy levels, which makes them quite stable.^8^ When excited by 335 nm wavelength light, the Ag NC ion population in the ground state S_0_ is promoted to the excited singlet state S_1_, following subsequently to the T state by a non-radiative singlet–triplet transition. Thereafter, the T state is depopulated by a radiative triplet–singlet transition to the ground state, which corresponds to the broad band emission centered around 500 nm.^26^ Also, a portion of the Ag NC ion population in the S_1_ state could populate the more energetic Eu^3+^ levels *via* an ET process. By means of non-radiative transition, the ^5^D_0_ level is populated, then transitions radiatively to the ^7^F_*j*_ providing narrow band emissions at the reddish-yellow region.

From the spectroscopic results, it is observed that different Ag species are formed during the melt-quenching and heat treatment processes. Similar results have been reported in the literature for Ag:Eu-co-doped oxyfluoride glasses.^8,30^ These studies propose the following reactions as a mechanism for precipitation of Ag nanoclusters and metallic Ag nano-particles.

2 Ag^+^ + e^−^ → Ag^0^

3 Eu^3+^ + e^−^ → Eu^2+^

4 Eu^2+^ + Ag^+^ → Eu^3+^ + Ag^0^

5 (*m* − *n*)Ag^0^ + *n*Ag^+^ → Ag^*n*+^_*m*_

Since the precipitation of Ag NPs was favored for higher europium concentrations in the co-doped samples after heat treatment, this behavior indicates that the Eu plays an important role in the formation processes of different Ag species. Dias *et al.*^22^ and Melo *et al.*^7^ reported that the incorporation of the RE oxides, Nd_2_O_3_ and Eu_2_O_3_, in the CaBAl glass composition leads to the conversion of BO_4_ into BO_3_ groups, forming a non-bridging oxygen (NBO). This conversion is more effective for higher RE concentrations. In addition, Simo *et al.*^31^ have reported that the electrons extracted directly from atoms that are intrinsic to the glass, *i.e.*, non-bridging oxygens (NBOs), may act as reductive agents in glasses.

Considering the statements above, two mechanisms are possible for the different Ag species formation in these glasses. The first one considers that the presence of NBOs induces the reduction of Ag^+^ → Ag^0^ and Eu^3+^ → Eu^2+^, reactions (2) and (3) respectively. However, these reactions must occur simultaneously to the reactions (4) and (5), so that the Eu^2+^ ions produced are immediately consumed, which causes a decrease in the concentration of Ag^+^ and Eu^2+^, justifying the non-significant evidence of divalent europium ions in the emission spectra and the reduction of the Ag^+^ excitation band. The second possible mechanism does not consider the Eu^3+^ reduction. In this way, only the reactions (2) and (5) must be occurring. As the number of NBOs increases due to Eu_2_O_3_ concentration, reaction (2) is also favored justifying a more intense formation of different Ag NCs and Ag NPs in the compositions with higher Eu_2_O_3_ amounts. Due to the almost absence of Eu^2+^ evidence in the PL results, we believe that the second mechanism is predominant in the CaBAl glass but, as mentioned previously, a possible Eu^3+^ and Eu^2+^ coexistence cannot be neglected. Thus, an additional characterization technique is needed to certify whether or not divalent Eu is present in the host. These characterizations will be carried out in future work.

The above spectral features, the emission spectra covering almost all the visible light region and the broadband excitation located at the UV region, indicate that Ag:Eu-co-doped CaBAL glasses have high potential for applications as white light phosphors for UV light excited W-LEDs.

### 3.3 Photoluminescence decay

**Fig. 5:**
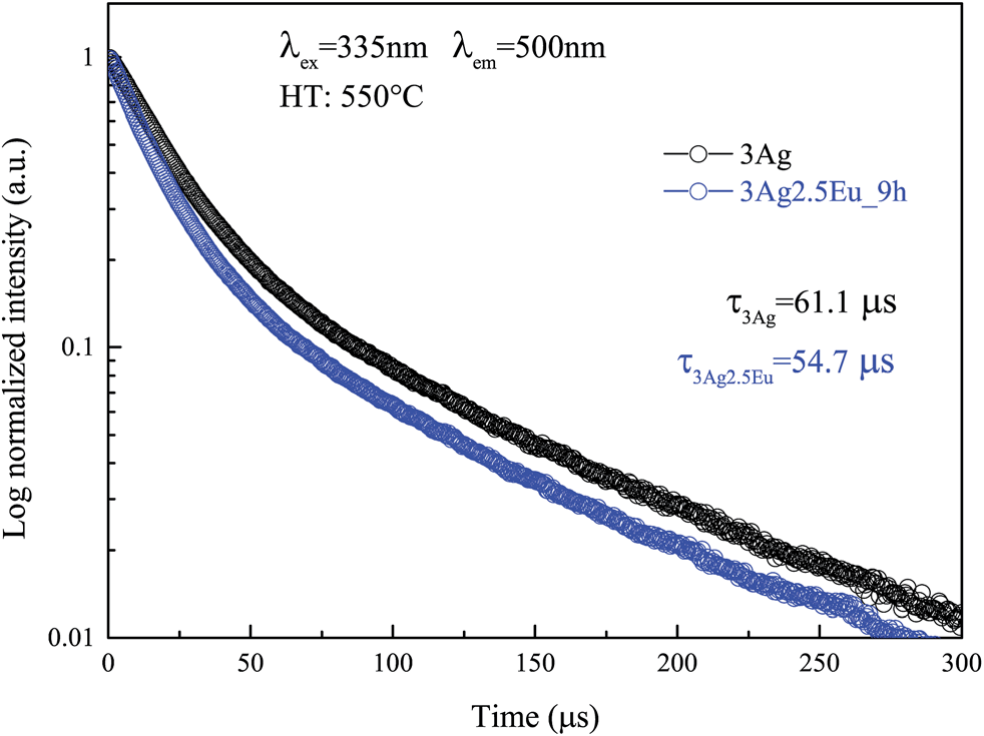
PL decay curves for 3Ag singly doped and 3Ag:2.5Eu-co-doped CaBAl glasses heat treated at 550 °C for 9 hours. Symbols are experimental data and continuous lines are the best curve fitted using eqn (6).

The PL decay can provide more information about ET processes from molecule-like Ag NCs to Eu^3+^. Fig. 5 shows the PL decay curve for Ag NC emission at 500 nm excited by 335 nm wavelength light. At first, independently to ET mechanisms, the decay kinetic was well fitted by a double exponential function given by6
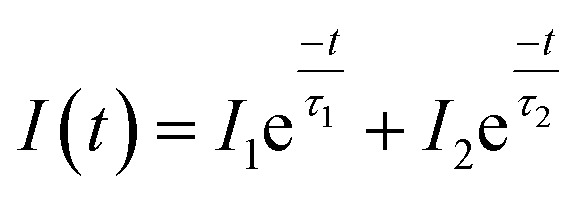
where *τ*_1_ and *τ*_2_ represent the short and long lifetime values, respectively. Thus, the mean lifetimes were obtained by7
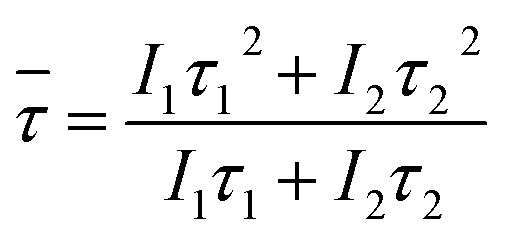


The Ag NC decay mechanism was explained by Tikhomirov *et al.* in a spectroscopic study of Ag nanoclusters in oxyfluoride glass.^26^ The authors reported that two non-equivalent Ag^2+^_4_ sites are predominantly formed in the lattice, which are distinguished by elongation along the diagonal of the Ag^2+^_4_ tetramer. Each one exhibits a different lifetime value, justifying the use of a double exponential function to fit. The mean lifetimes were evaluated to be about 61.1 μs for the 3Ag singly doped sample and 54.7 μs for the 3Ag:2.5Eu-co-doped sample annealed at 550 °C for 9 hours.

The short (*τ*_1_), long (*τ*_2_) and mean lifetime (*τ̄*) values for the CaBAl glasses are presented in Table 1. Both lifetime components of the excited Ag NCs (donor) exhibit a value decrease in the presence of Eu^3+^ ions (acceptor). This lifetime decrease is evidence that the improved luminescence is due to the energy transfer process Ag NCs → Eu^3+^ instead of the local-field SRP effect.

**Table 1:** Experimental lifetime components, short (*τ*_1_), long (*τ*_2_) and mean (*τ̄*) for the Ag singly doped and Ag:Eu-co-doped CaBAl glasses, untreated and heat treated at 550 °C for 9 hours

	*τ*_1_ (μs)	*τ*_2_ (μs)	*τ̄* (μs)
3Ag	19.5 ± 0.2	93.1 ± 0.1	61.1 ± 0.6
3Ag1Eu	15.6 ± 0.2	90.7 ± 0.1	60.8 ± 0.6
3Ag1Eu-9 h	15.1 ± 0.1	88.7 ± 0.1	57.9 ± 0.6
3Ag2.5Eu	15.6 ± 0.2	86.4 ± 0.1	57.6 ± 0.6
3Ag2.5Eu-9 h	15.5 ± 0.2	84.6 ± 0.1	54.7 ± 0.5

### 3.4 CIE color coordinates

As previously mentioned, Ag:Eu-co-doped CaBAl glasses have high potential for applications as white light emission phosphors. The excitation spectra show that these glasses are excited by a wide wavelength range. Thus, to further evaluate their applicability in WL emission devices, the photoluminescence spectra were acquired under a violet LED excitation (405 nm). The color coordinates were determined by deconvolving the combined LED and sample emissions using three color matching functions, which are established by the Commission Internationale de l’Eclariage (CIE).

**Fig. 6:**
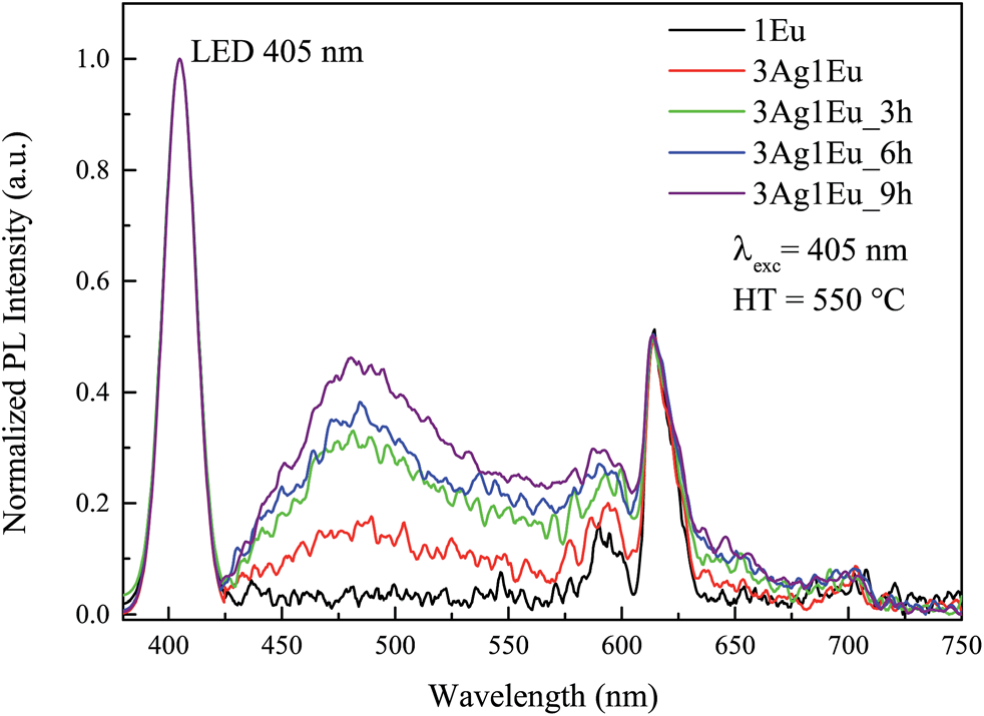
Normalized emission spectra of Eu singly doped and 3Ag:1Eu-co-doped CaBAl glasses under 405 nm excitation combined with simulated LED emission for different HT times at 550 °C.

Fig. 6 shows the normalized emission spectra of Eu singly doped and 3Ag:1Eu-co-doped samples under 405 nm excitation combined with a simulated LED emission. Besides the Eu singly doped sample, all co-doped samples exhibit a broadband emission covering the entire visible spectrum. The band centered at about 480 nm increases with longer HT time, in agreement with the results previously presented in Fig. 3. This is due to the Ag NC concentration increase being promoted by the HT process. The same behavior was observed for the 3Ag:2.5Eu samples heat treated at 550 °C (not shown), on the other hand, these samples when heat treated at 575 °C show a quenching effect similarly to the previous results under 355 nm excitation (Fig. 3).

**Fig. 7:**
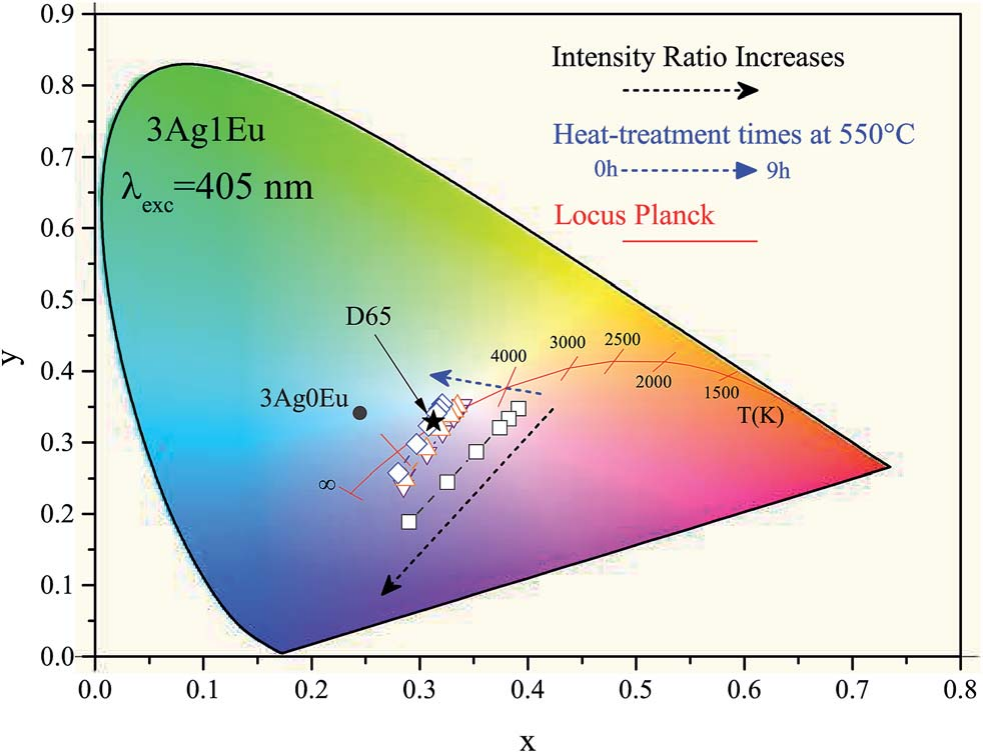
CIE-1931 diagram for 3Ag:0Eu (filled circle) and 3Ag:1Eu-co-doped CaBAl glasses combined with simulated 405 nm LED emission for different HT times at 550 °C (open symbols) and D65 illuminant (filled star).

To evaluate the viability of achieving an ideal WL, the emitting colors were evaluated by adjusting the luminescence intensity ratio between the violet LED (*I*_L_) and the glass phosphors (*I*_G_). The intensity ratio was set at *I*_L_/*I*_G_ = 0, 1, 2, 5, 10 and 20. In Fig. 7, the corresponding *x*–*y* color coordinates of 3Ag:0Eu (filled circle) and 3Ag:1Eu samples for different HT times at 550 °C (open symbols) are depicted in the CIE-1931 diagram. The black arrows indicate the ratio *I*_L_/*I*_G_ increase and the blue arrows indicate the longer HT times. After performing a HT process, the color coordinates are shifted from the reddish to the blue-green region, approaching a white color. Also, the color coordinates of the D65 light source (0.31, 0.33) (filled star) with a correlated color temperature (CCT) at 6504 K are indicated as reference. D65 is a standard illuminant, recommended by the CIE, representing the average daylight in both visible and UV regions.^32^

**Fig. 8:**
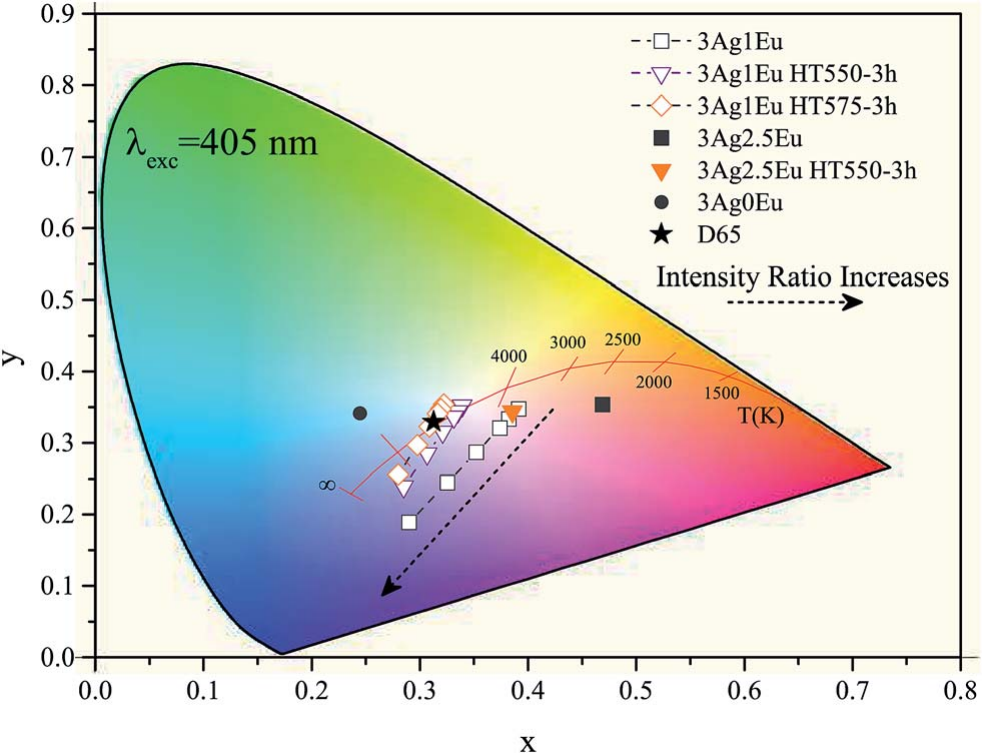
CIE-1931 diagram for: 3Ag:0Eu (filled circle), and 3Ag:1Eu-co-doped CaBAl glasses combined with simulated 405 nm LED emission, when non heat treated, and treated at 550 and 575 °C (open symbols), 3Ag:2.5Eu (filled symbols) and D65 illuminant standard (filled star).

In Fig. 8, the color coordinates of the 3Ag:1Eu samples non heat treated, treated at 550 and 575 °C for 3 hours are shown. Higher temperature treatment also leads to a slight color coordinate shift to the blue-green region. Samples with a higher europium concentration (2.5 wt%) have color coordinates that are more shifted towards the red region, because the characteristic emission of Eu^3+^ ions in the red region is more intense for these samples, which is not intended for applications in WL devices.

It is important to emphasize that the 3Ag:1Eu heat treated samples present color coordinates very close to the ideal white coordinate (0.33, 0.33), as illustrated in Fig. 7 and 8. In addition, different emission colors can be obtained by adjusting the *I*_L_/*I*_G_ ratio value. For example, samples treated at 550 °C for 9 h and at 575 °C for 3 h when the LED emission intensity is 5 times greater than the sample emission had resulting color coordinates of [(0.31, 0.32); CCT 6825K] and [(0.31, 0.33); CCT 6772K], respectively, practically equal to the D65 coordinates.

**Fig. 9:**
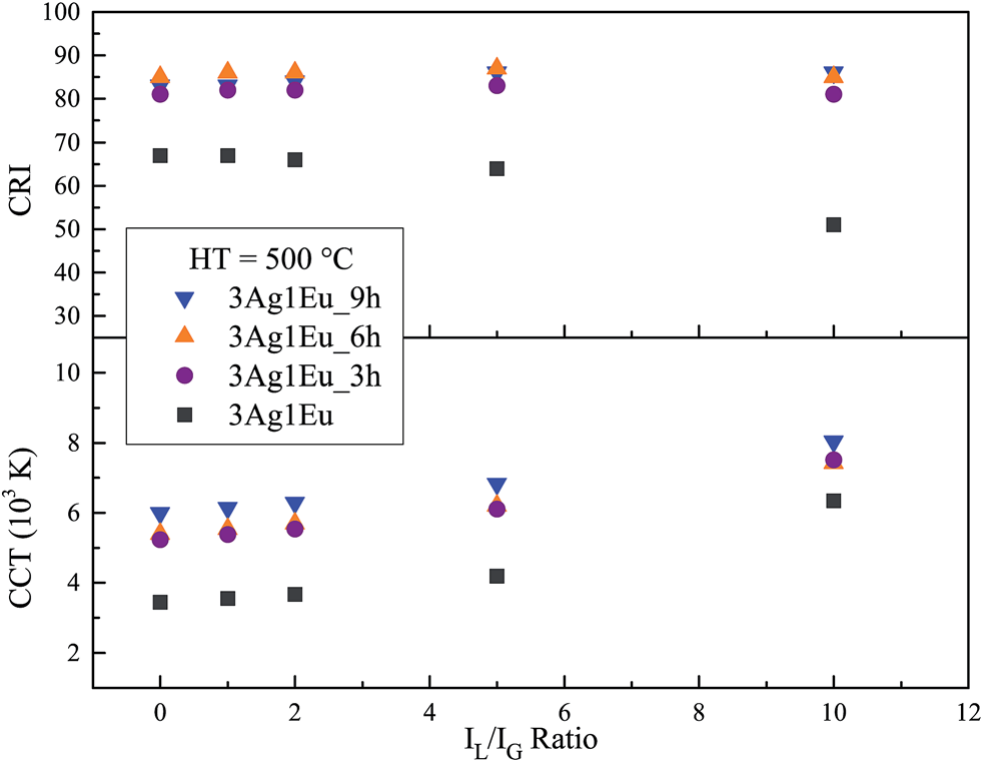
CRI and CCT (K) as a function of *I*_L_/*I*_G_ ratio for 3Ag:1Eu-co-doped CaBAl glasses HT at 550 °C.

The CCT of 3Ag:1Eu samples heat treated at 550 °C was calculated as a function of *I*_L_/*I*_G_ ratio and the results are shown in Fig. 9, which also shows that the HT process leads to a CCT increase. In addition, a wide range of emission color temperatures can be achieved by the adjustment of the intensity *I*_L_/*I*_G_ ratio, which allows reproduction of the variations of sky light spectral distribution. Therefore, a route for smart lighting can be achieved by the adjustment of LED intensity, demonstrating a good agreement to the human circadian cycle.

Another important color quality indicator for white light emitting devices is the CIE color rendering index (CRI). The CRI is a measure from 0 to 100 that indicates the power of a light source to reveal the real color of objects in comparison with a natural light. Details about the CRI calculation method can be found in ref. 32. The CRI values were calculated as a function of *I*_L_/*I*_G_ ratio for 3Ag:1Eu 550 °C heat treated samples and results are also shown in Fig. 9. The CRI results achieved for samples HT for 6 h and 9 h are above 80. These results are very high and are comparable with the standard illuminant D65 (CRI = 100) suggested by the CIE.^32^

## 4 Conclusion

Ag/Eu singly doped and co-doped calcium boroaluminate glasses were prepared by a melt-quenching method. Heat treatment effects on the formation processes of different Ag species and their influence on the glass photoluminescence properties have been studied. The absorption spectra indicate a successful HT-induced Ag NP precipitation in the host due to the emergence of a SPR absorption band. Results from PL spectroscopy also indicate the presence of Eu^3+^ ions and the formation of the luminescent species, Ag^+^ and molecule-like Ag (Ag NCs). A new excitation band, around 335 nm, was observed for the co-doped samples monitoring the Eu^3+^ radiative transition at 614 nm, which suggests an energy transfer route from the Ag species (donor) to the Eu^3+^ (acceptor). A significant intensity increase in Eu^3+^ emission and a PL lifetime decrease for Ag NC emission were observed under 335 nm excitation, reinforcing the assumption of an energy transfer process. Thus, in the context of understanding the metal–RE interaction we can conclude that, when incorporated in the CaBAl glass, the enhanced luminescence is due to the energy transfer process Ag NC → Eu^3+^ instead of the local-field SPR effect from the Ag nanoparticles. In this case the Ag NPs provide a PL quenching effect.

Ultimately, by combining the Ag NCs bright bluish-white emission and the intense Eu^3+^ red emission with a violet LED emission, the convolved luminescence can be adjusted in order to obtain the ideal WL compared to the CIE color coordinates. Also, a route for simulating the sky light variations can be provided, close to the circadian light cycle. The color results characterize the Ag:Eu-co-doped CaBAl glasses as great potential phosphors for white light emitting devices.

## Conflicts of interest

There are no conflicts to declare.

## Supplementary Material
